# CR1 clade of non-LTR retrotransposons from *Maculinea *butterflies (Lepidoptera: Lycaenidae): evidence for recent horizontal transmission

**DOI:** 10.1186/1471-2148-7-93

**Published:** 2007-06-25

**Authors:** Olga Novikova, Ewa Śliwińska, Victor Fet, Josef Settele, Alexander Blinov, Michal Woyciechowski

**Affiliations:** 1 Laboratory of Molecular Evolution, Institute of Cytology and Genetics SB RAS, Novosibirsk, Russia; 2 UJAG – Jagiellonian University, Institute of Environmental Sciences, Krakow, Poland; 3 Marshall University, Huntington, West Virginia, USA; 4 Department of Community Ecology, UFZ – Centre for Environmental Research Leipzig-Halle, Halle (Saale), Germany

## Abstract

**Background:**

Non-long terminal repeat (non-LTR) retrotransposons are mobile genetic elements that propagate themselves by reverse transcription of an RNA intermediate. Non-LTR retrotransposons are known to evolve mainly via vertical transmission and random loss. Horizontal transmission is believed to be a very rare event in non-LTR retrotransposons. Our knowledge of distribution and diversity of insect non-LTR retrotransposons is limited to a few species – mainly model organisms such as dipteran genera *Drosophila*, *Anopheles*, and *Aedes*. However, diversity of non-LTR retroelements in arthropods seems to be much richer. The present study extends the analysis of non-LTR retroelements to CR1 clade from four butterfly species of genus *Maculinea *(Lepidoptera: Lycaenidae).

The lycaenid genus *Maculinea*, the object of interest for evolutionary biologists and also a model group for European biodiversity studies, possesses a unique, specialized myrmecophilous lifestyle at larval stage. Their caterpillars, after three weeks of phytophagous life on specific food plants drop to the ground where they are adopted to the ant nest by *Myrmica *foraging workers.

**Results:**

We found that the genome of *Maculinea *butterflies contains multiple CR1 lineages of non-LTR retrotransposons, including those from MacCR1A, MacCR1B and T1Q families. A comparative analysis of RT nucleotide sequences demonstrated an extremely high similarity among elements both in interspecific and intraspecific comparisons. CR1A-like elements were found only in family Lycaenidae. In contrast, MacCR1B lineage clones were extremely similar to CR1B non-LTR retrotransposons from Bombycidae moths: silkworm *Bombyx mori *and *Oberthueria caeca*.

**Conclusion:**

The degree of coding sequence similarity of the studied elements, their discontinuous distribution, and results of divergence-versus-age analysis make it highly unlikely that these sequences diverged at the same time as their host taxa. The only reasonable alternative explanation is horizontal transfer. In addition, phylogenetic markers for population analysis of *Maculinea *could be developed based on the described non-LTR retrotransposons.

## Background

Non-long terminal repeat (non-LTR) retrotransposons are mobile genetic elements that propagate by reverse transcription of an RNA intermediate. These elements lack terminal repeats and utilize a simplified target-primed reverse transcription (TPRT) mechanism for their retrotransposition. During TPRT, the element-encoded endonuclease cleaves the genomic DNA, and reverse transcriptase (RT) uses this break to prime reverse transcription from the element's RNA. Resulting cDNA copy is then integrated into the target site [1,2].

Based on their structure, non-LTR retrotransposons can be classified into two groups. The first group has a single open reading frame (ORF) that encodes RT in the middle and a restriction enzyme-like endonuclease (RLE) near its C-terminus. The second group of non-LTR retrotransposons has two ORFs: ORF1 and ORF2; the latter encodes two domains responsible for retrotransposition: apurinic/apyrimidinic endonuclease (APE)-like endonuclease domain at the N-terminus and RT domain in the middle.

The RT domain has been used to classify non-LTR retrotransposons into phylogenetic groups, or clades [3]. Originally, 11 clades were distinguished among non-LTR retrotransposons. Later, the total number of clades increased to 16, with the addition of Genie [4], NeSL-1 [5], Ingi, Rex1 [6], and L2 clades [7]. This number is likely to increase further since almost every detailed study of non-LTR retrotransposons brings additional information about their phylogeny and diversity [8-10]. For example, a new family encoding both RLE and APE endonucleases was described recently [10].

Non-LTR retrotransposons represent a large fraction of known retroelements in insects. At the same time, most of our knowledge on distribution and diversity of non-LTR retrotransposons from insects is limited to model organisms such as dipteran genera *Drosophila*, *Anopheles*, and *Aedes *[8,11,12]. Almost nothing is known about many other insect groups. Several studies attempted to test a wide range of arthropods for the presence of particular clades, e.g. distribution of R1 and R2 clades has been explored in detail in different arthropods [13].

Our recent study of scorpions (Arachnida) revealed an unexpected diversity of non-LTR retrotransposons within several clades in this ancient arthropod group, with at least three distant clusters in CR1 clade [14]. Previously, only one family, T1Q-like elements, was described inside this clade from arthropods [3,8,11]. Studies of non-LTR retrotransposon diversity can provide a valuable contribution into understanding of evolution and spreading of mobile elements in arthropods.

The mode of retrotransposition and phylogenetic studies suggested that evolution of non-LTR retrotransposons proceeds mainly via vertical transmission and random loss of elements from a population [3]. Horizontal transmission is believed to be a very rare event for this class of retrotransposons. Nevertheless, at least one well-documented case of horizontal transmission is known. Horizontal transmission of Bov-B elements from an ancestral snake lineage (Boidae) to the ancestor of ruminants has been confirmed on the basis of discontinuous distribution, extreme nucleotide sequence conservation, and phylogenetic analysis [15].

In the present study, we examined the diversity of CR1 clade of non-LTR retrotransposons in lycaenid butterflies of the genus *Maculinea*. Molecular systematic studies confirmed the existence of seven *Maculinea *species, most of them with several subspecies [16]. *Maculinea arion *L., *M. alcon *(Denis et Schiffermüller), and *M. teleius *(Bergsträsser) are widely distributed from western Europe to East Asia, while *M. nausithous *(Bergsträsser) ranges from West Europe to Central Asia. *M. cyanecula *(Eversmann) is restricted to Central Asia, while *M. arionides *(Staudinger) and *M. kurenzovi *(Sibatani, Saigusa *et *Hirowatari) are found in East Asia [16-18]. In Europe, *Maculinea *are an object of interest of evolutionary biologists and model species for biodiversity studies since they are considered vulnerable or threatened [19-21] and also because of their fascinating biology. All *Maculinea *species are characterized by a specialized myrmecophilous lifestyle at larval stage. Their caterpillars, after three weeks of phytophagous life on specific food plants drop to the ground where they are adopted to the ant nest by *Myrmica *foraging workers. Larvae remain within the nest for a period of approximately 10 or 22 months, during which time they increase their body mass almost 100 times without moulting [22-24]. Three *Maculinea *species, *M. teleius*, *M. nausithous *and *M. arion*, are obligatory predators of the ant larvae. However, *M. alcon*, so-called "cuckoo" species, is a fully integrated social parasite and is fed by workers by trophic eggs and regurgitation [25].

Their unusual life history traits, such as parasitic life style, differentiated feeding strategies, exceptions to normal insect growth rules as an adaptation to long starvation period [26] and one or two-year mode of caterpillar's growth, make genus *Maculinea *a very interesting model for evolutionary studies, including studies of non-LTR retrotransposons diversity and evolution. Moreover, non-LTR retrotransposons could be very useful as molecular markers in intraspecific phylogeography of *Maculinea*.

We implemented broad analysis for CR1 group in an attempt to cover the diversity of CR1-like elements from *Maculinea *butterflies and evaluate the possibilities for development of phylogenetic markers. Distinct evolutionary lineages of the CR1-like non-LTR retrotransposons were identified, showing significant variation among CR1 clade elements.

Surprisingly, retroelements highly similar to one of the detected CR1 families have been recently identified in silkworm *Bombyx mori*. We reconstructed the complete element BmCR1B from *B. mori *using the genomic fragments available in databases. Extremely high similarity of *B. mori *and *Maculinea *elements was intriguing since these lepidopteran species are not closely related. We performed additional PCR screening of butterflies from distinct groups (suborder Ditrysia) to evaluate distribution of newly isolated families from CR1 clade. CR1A-like elements were found only in family Lycaenidae. In contrast, CR1B-like non-LTR retrotransposons were detected in Bombycidae moths: silkworm *Bombyx mori *and *Oberthueria caeca*. The degree of coding sequence similarity of the studied elements, their discontinuous distribution and results of divergence-versus-age analysis make it highly unlikely that these sequences diverged at the same time as their host taxa. Thus, new evidence was obtained for horizontal transmission of non-LTR retrotransposons.

## Results and discussion

### Multiple lineages of CR1-like retroelements represent in *Maculinea*

No retrotransposable elements from *Maculinea *have been previously described. The degenerate oligonucleotide primers used in our study were designed based on sequences of known non-LTR retrotransposons from CR1 clade. Four species of *Maculinea *were screened by PCR using these degenerate primers (Table 1), and the PCR products were cloned.

**Table 1:** *Maculinea *species used in this study, lineages detected in CR1 clade, and GenBank accession numbers of isolated clones.

genus	species	lineage	No. of clones with RT	Accession numbers
*Maculinea*	*teleius*		15	
		lineage A	7	[GenBank:DQ823008],
				[GenBank:DQ823009],
				[GenBank:DQ823010],
				[GenBank:DQ823011],
				[GenBank:DQ823016],
				[GenBank:DQ823017],
				[GenBank:DQ823018]
		lineage B	6	[GenBank:DQ823029];
				[GenBank:DQ823030];
				[GenBank:DQ823035],
				[GenBank:DQ823036],
				[GenBank:DQ823037],
				[GenBank:DQ823038]
		lineage C	2	[GenBank:DQ836363];
				[GenBank:DQ836364]
*Maculinea*	*nausithous*		13 (+1)*	
		lineage A	8	[GenBank:DQ823012],
				[GenBank:DQ823013],
				[GenBank:DQ823014],
				[GenBank:DQ823019],
				[GenBank:DQ823020],
				[GenBank:DQ823021],
				[GenBank:DQ823022],
				[GenBank:DQ823023]
		lineage B	3	[GenBank:DQ823031];
				[GenBank:DQ823032];
				[GenBank:DQ823039]
		lineage C	2	[GenBank:DQ836365];
				[GenBank:DQ836366]
*Maculinea*	*alcon*		10 (+1)*	
		lineage A	8	[GenBank:DQ823012];
				[GenBank:DQ823024],
				[GenBank:DQ823025],
				[GenBank:DQ823026],
				[GenBank:DQ823027],
				[GenBank:DQ823028]
		lineage B	2	[GenBank:DQ823033];
				[GenBank:DQ823034]
*Maculinea*	*arion*		9	
		lineage A	6	[GenBank:DQ994657],
				[GenBank:DQ994658],
				[GenBank:DQ994659],
				[GenBank:DQ994660],
				[GenBank:DQ994661],
				[GenBank:DQ994662]
		lineage B	3	[GenBank:DQ994663],
				[GenBank:DQ994664],
				[GenBank:DQ994665]

* single clones appeared to be R1 elements

In total, 52 clones were obtained for CR1 clade and sequenced (Table 1). After preliminary identification of RT fragments by comparison with sequences in the GenBank database, we found that 49 clones showed clear similarity with RT domain of non-LTR retrotransposons, while three sequences revealed no presence of RT. The total number of clones with RT for each species is listed in Table 1.

The BLAST search showed a strong similarity of translation products of those clones that contained RT to the elements described from CR1 clade. In general, the designed PCR primers for CR1 proved to be suitable for isolation of an RT domain from *Maculinea*. However, two sequences unexpectedly showed clear similarity to the elements belonging not to CR1 but to R1 clade of non-LTR retrotransposons. These sequences were named MnaR1 (*Maculinea nausithous *R1 element) [GenBank:DQ836391] and MalR1 (*Maculinea alcon *R1 element) [GenBank:DQ836392]. Representatives of R1 clade appear to play a very important role in telomere maintenance in insects [27,28]. For example, TRAS1 and SART1 elements from *Bombyx mori *are site-specific for telomeric repeats [29]. Two isolated *Maculinea *elements shared more than 57 % homology in amino acid sequence and were highly similar to R1-like elements from other insects. Similarity between amino acid sequences of MnaR1 and RT1 element from *Anopheles gambiae *[30] was 51 %. Amplification of R1 elements using CR1 pair of primers could possibly be a result of primer degeneracy.

The majority of detected CR1-like elements revealed defective translated products (29 clones). In total, 18 elements represented putatively intact RT sequences. Of 29 defective clones, 15 has a single stop mutation and no frameshifts, while 14 clones contained variable-length indels and resulted in frameshifts.

**Figure 1:**
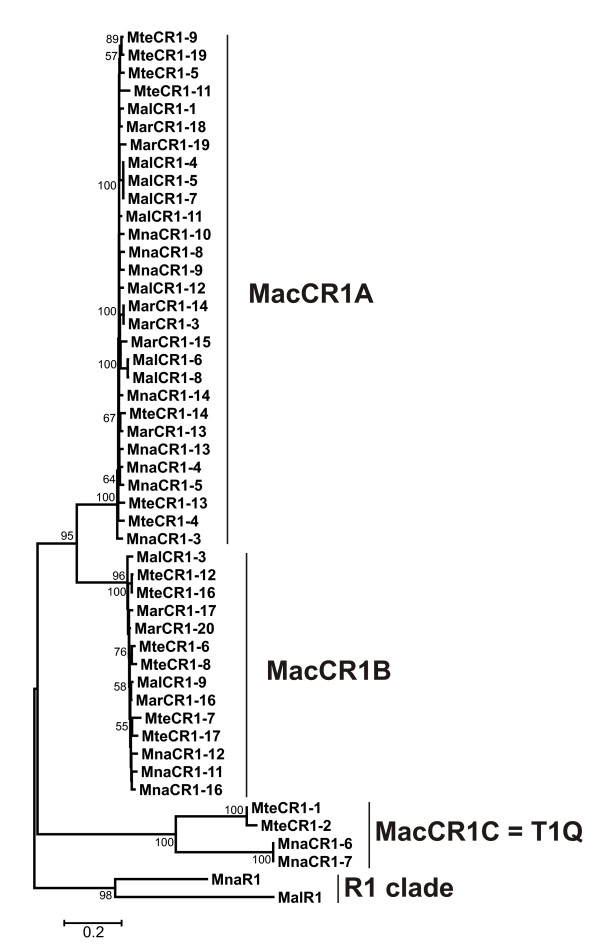
**NJ phylogeny based on partial RT domain nucleotide sequences of newly isolated retrotransposons from four species of *Maculinea***. Statistical support was evaluated by bootstrapping (1000 replications); nodes with bootstrap values over 50% are indicated. CR1 elements can be divided into three distinct lineages: MacCR1A, MacCR1B and MacCR1C.

We conducted both intra- and interspecific comparative analysis of RT nucleotide sequences. Presence of several distinct groups was detected (data not shown). The phylogeny was constructed based on nucleotide sequences of all 49 isolated clones. Neighbor-joining (NJ) analysis demonstrated presence of three clear lineages (Figure 1). Two most commonly found types of CR1-like elements in *Maculinea *were designated Lineage A (MacCR1A) and Lineage B (MacCR1B) (see Figure 1; Additional file 1).

### Lineage A of CR1-like elements from *Maculinea *– MacCR1A family

In total, 29 clones from all four studied species formed this lineage in NJ analysis based on nucleotide sequences of RT domain (Figure 1). Comparative analysis of RT nucleotide sequences showed an extremely high similarity among elements both within and between species. Average intraspecific sequence similarity ranged from 98.6 % in *M. alcon *to 99.5 % in *M. teleius*. Less than 2 % of intraspecific divergence was detected in *M. nausithous*. Three of eight clones isolated from *M. alcon *appeared to be absolutely identical (clones MalCR1-4, 5, and 7 - *Maculinea alcon *CR1 clones 4, 5, and 7).

The potential translation products of the sequences were determined. Eleven clones contained intact RT sequences and could represent the master sequences of elements with RT activity. Eight of isolated elements had stop mutations but no frameshifts. The remaining ten clones contained indels in comparison with the consensus sequence. Indels resulted in frame-shifts in five elements. All indels seems to be unique for a specific element copy since single copies were detected for each case of insertion or deletion.

Kimura-corrected distances were calculated within and between species using 14 selected copies (only one copy was used for the identical MalCR1-4, 5, and 7). The average distance was 3.1% within *M. teleius*, 2.7% within *M. nauthitous*, and 3.6% within *M. alcon*. Interspecific distances ranged from 3.1% between *M. teleius *and *M. nausithous *to 3.6% between *M. teleius *and *M. alcon*. These low Kimura-corrected distances indicate relatively recent retrotransposition events for Lineage A elements.

Phylogeny of *Maculinea *based on nucleotide sequences of CR-1-like elements was not congruent with the known phylogeny of host species (Figure 1). Moreover, no relationships could be reconstructed within Lineage A elements. Altogether, 100 variable sites per 553 bp were detected among aligned nucleotide sequences, and 49 variable amino acid residues per 184 sites of amino acid sequence alignment. However, only six variable sites were parsimoniously informative at DNA level, and only two, at amino acid level. Other variable sites were represented by singletons and were non-informative for phylogenetic analysis.

### Lineage B of CR1-like elements from *Maculinea *– MacCR1B family

Lineage B of CR1-like elements from *Maculinea *butterflies was represented by 14 clones. The phylogenetic tree based on NJ analysis of nucleotide sequences showed a strong support for separation of this lineage from elements of the Lineage A (bootstrap 100 %). However, the topology within Lineage B was not supported.

A comparative analysis of nucleotide and amino acid sequences within the Lineage B demonstrated high similarity both within and among species. Two clones from *M. teleius *(MteCR1-12 and MteCR1-16) appeared to be identical; both carried 11 bp insertion and 40 bp deletion. One more clone MteCR1-7 contained 17 bp insertion and 32 bp deletion. The remaining sequences had no indels; however, two of them (MteCR1-17 and MnaCR1-11) represented interrupted translated products. Six sequences seemed to be the copies of putatively intact retrotransposons, with the average genetic distance less than 2 %. The most distant copy (MteCR1-6) showed nucleotide sequence divergence 4.1 %.

For phylogenetic analysis, we selected eight copies without compensatory frameshift mutations. Nucleotide and amino acid sequences were aligned. Variable and parsimoniously informative sites were determined for both alignments. As in MacCR1A lineage, number of parsimoniously informative sites was very low. Among 66 variable sites per 547 bp of sequence only eight were informative for phylogenetic analysis while 58 substitutions were copy-specific. Number of sites with variable amino acid residues was less than in the Lineage A, only 36 sites per 182 amino acid residues. Four parsimoniously informative sites were detected, compared to two in lineage A; such low number of informative sites does not allow meaningful phylogeny reconstruction among lineage B. Low Kimura-corrected distances within and among species (average 3.3% in lineage B) suggest a relatively recent retrotransposon activity.

MacCR1A and MacCR1B elements were only 73.5 % similar at DNA level but 88.5 % similar at amino acid level.

### Lineage C – T1Q-like non-LTR retroelements from *Maculinea*

Only four retroelements from lineage C were isolated from two species: *M. teleius *and *M. nausithous *(Figure 1). MnaCR1-6 and MnaCR1-7 showed 100 % similarity, whereas MteCR1-1 and MteCR1-2 differ in length and contain two substitutions per 411 bp of common sequence. MteCR1-1 sequence is 546 bp long; MteCR1-2 is truncated at 5' portion and 411 bp long. Translated products of MteCR1-2 and MteCR1-1 contain no stop mutations or frameshifts and seem to be putatively intact. MnaCR1-6 and MnaCR1-7 clones contained not only stop codons but also frameshifts. We reconstructed the reading frame for MnaCR1-6/7 element. Comparison of amino acid sequences showed 66.3 % similarity between MteCR1-1 and reconstructed MnaCR1-6/7, but only 55.9 % similarity was found at the DNA level.

Elements from MacCR1C lineage share high similarity with T1 and Q elements and seem to belong to the T1Q lineage [3,31,32]. The amino acid sequence similarity ranged from 53.3 % between MnaCR1-6/7 and Q element to 54.7 % between MteCR1-1 and Q element; the isolated elements were less than 50% similar to the CR1 retrotransposon from chicken *Gallus gallus *[33]. The T1Q lineage was detected from genomes of two out of four studied species of *Maculinea*. One element was isolated from *M. nausithous*, and two closely related copies, from *M. teleius*. Small fraction of T1Q-like elements among isolated clones could be due to a very low copy numbers of this lineage in the studied species. It could be also a consequence of high divergence among the elements in the sequences annealing to PCR primers.

### BmCR1B element from *B. mori*

The nucleotide and amino acid sequences of MacCR1A and MacCR1B elements were used as queries in BLAST search against databases for arthropods. No species among those available in databases gave the positive match except of silkworm *Bombyx mori*. MacCR1B was found to be present in *B. mori *genome. Surprisingly, CR1B elements from *Maculinea *and *B. mori *showed extremely high sequence similarity.

**Figure 2:**
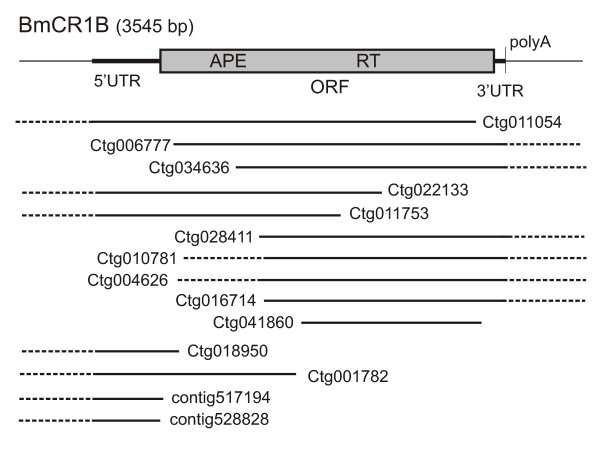
**The structural organization of BmCR1B retroelement from *B. mori *reconstructed based on genomic fragments available in databases**. The numbers of respective contigs are given. Single open reading frame (ORF) was found which codes protein carried reverse transcriptase (RT) and endonuclease (APE) domains. BmCR1B element has 5'- and 3'- untranslated terminal regions (5' and 3' UTR) and polyadenine tract (polyA) at the 3' end.

Based on BLAST results, we reconstructed non-LTR retrotransposon from *B. mori*, named BmCR1B (*Bombyx mori *CR1 element family B). We did not find any genomic fragments containing the entire sequence of BmCR1B element. However, partial BmCR1B sequences from different genomic fragments showed high similarity to each other and the entire sequence of the element could be assembled based on several contigs (Figure 2).

BmCR1B element could be almost completely reconstructed by fusion of two contigs: Ctg011054 [GenBank:AADK01011054] and Ctg006777 [GenBank:AADK01006777]. The fragment Ctg011054 lacks approximately 400 bp of element at the 3' end. The second contig, Ctg00677, represents almost entire element BmCR1B but lacks 400 bp at 5' end. Other contigs contain incomplete sequences of BmCR1B retroelement to a greater extent, either due to their position at a contig border (Ctg034636; Ctg022133; Ctg011753; Ctg028411; Ctg016714) or to insertion of sequences of an unknown origin (Ctg010781 and Ctg004626).

**Figure 3:**
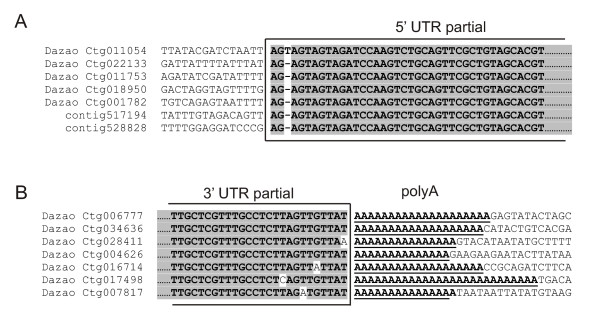
**Reconstruction of BmCR1B retroelement from *Bombyx mori***. Putative sequences of (A) 5' untranslated terminal region (UTR) and (B) 3' untranslated terminal region based on sequences of several genomic fragments. Polyadenine tract (polyA) is also shown.

The 3' end of the BmCR1B element was identified easily since it was terminated by polyA track. For reconstruction of the 5' end of element we performed additional BLAST search using as a query that sequence, which was homologous to Ctg011054, Ctg022133 and Ctg011753 in their 5' parts. Analysis of the 5' part of the BmCR1B from different contigs allowed to identify clearly the 5' end of the element (Figure 3). We expected that the full-length retroelement will be at least 5 Kb in length and will contain two ORFs. However, reconstructed BmCR1B elements are approximately 3550 bp in length and contain a single putative ORF, which encodes a polyprotein with APE and RT domains. PolyA tail with various lengths was detected at the 3' end of these elements (Figure 3). The putative 5' untranslated region (5' UTR) is 312 bp long. The putative 3' UTR is very short, only 53 bp.

**Figure 4:**
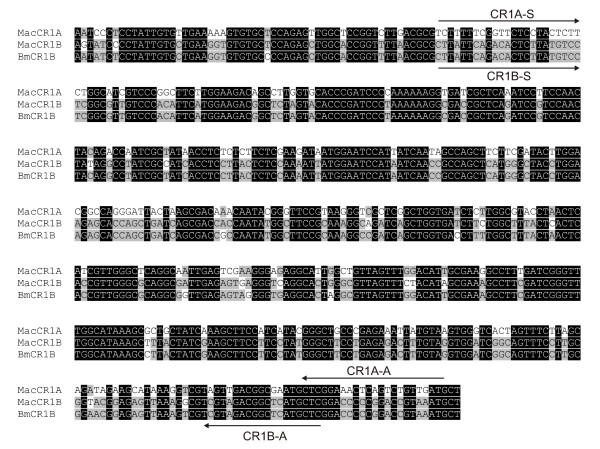
**Alignment of partial MacCR1A, MacCR1B and BmCR1B nucleotide sequences**. Location of primers specific for CR1A (CR1A-S and CR1A-A) and CR1B (CR1B-S and CR1B-A) families are shown.

The fragments of BmCR1B element detected in different contigs are highly similar to each other as well as to MacCR1B elements. Sequence similarity between reconstructed BmCR1B and consensus MacCR1B was 96.1 % at DNA level and 95 % at amino acid level. Similarity between BmCR1B element and consensus MacCR1A was 73.5 % at DNA level and 89 % at amino acid level (Figure 4).

### Distribution of CR1B and CR1A families in other butterflies

We designed specific PCR primers for both discovered families (MacCR1A and MacCR1B) and screened a selection of species from other related groups of Lepidoptera. Primers were based on sequences isolated from *Maculinea *spp. and BmCR1B element (Figure 4) and tested by PCR and sequencing in all four species of *Maculinea*. The primers annealed specifically either to MacCR1A or to MacCR1B template.

**Figure 5:**
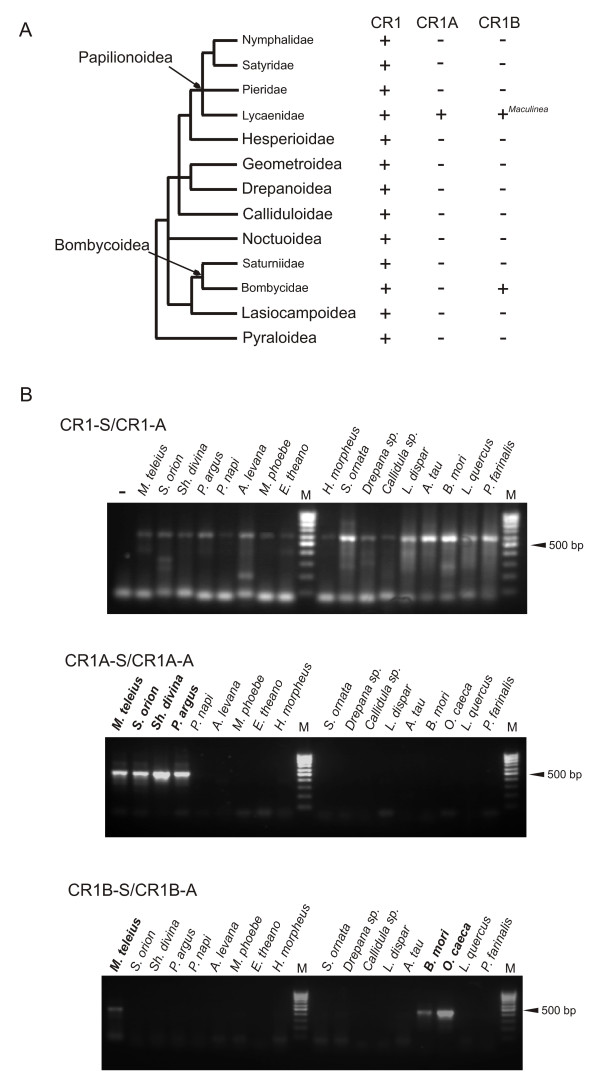
**Distribution of CR1B and CR1A families in Lepidoptera based on PCR amplification**. Phylogeny of studied superfamilies and families of butterfly suborder Ditrysia according to [34], with modifications (A). Families within Papilionoidea and Bombycoidea are shown since these superfamilies were analyzed in more detail. (B) PCR amplification of lepidopteran species with the primer pairs CR1-S/CR1-A (expected amplicon length 550 bp), CR1A-S/CR1A-A (expected amplicon length 520 bp) and CR1B-S/CR1B-A (expected amplicon length 520 bp) resolved in 1.3% agarose gel and stained with ethidium bromide. Lane "-", negative control; lane M, 100 bp DNA ladder.

Nine superfamilies and 13 families of Lepidoptera were selected based on known phylogeny of suborder Ditrysia including superfamily Bombycoidea (Table 2; Figure 5). Eight species from superfamily Papilionoidea were also studied. We included in our analysis three additional species of Lycaenidae since we expected to detect either both families or one of them in the genera closely related to *Maculinea*. Moreover, representatives of three other families from Papilionoidea were screened. We also chose one member of the Saturniidae, a bombycoid family closely related to the Bombycidae. As an outgroup served the meal moth *Pyralis farinalis *(superfamily Pyraloidea), the most ancient taxon among studied Lepidoptera according to the commonly accepted phylogeny [34]. *Oberthueria caeca *was included as additional species from family Bombycidae.

**Table 2:** List of Lepidoptera species used in present study in addition to *Maculinea *spp. Results of PCR screening with degenerate primers for CR1 clade, and specific primers for CR1A and CR1B families.

superfamily	family	species	CR1	CR1A	CR1B
Papilionoidea	Lycaenidae	*Scolitantides orion*	+	+	-
		*Shijimaeoides divina*	+	+	-
		*Plebejus argus*	+	+	-
	Pieridae	*Pieris napi*	+	-	-
	Nymphalidae	*Araschnia levana*	+	-	-
		*Melitaea phoebe*	+	-	-
	Satyridae	*Erebia theano*	+	-	-
Hesperioidea	Hesperiidae	*Heteropterus morpheus*	+	-	-
Geometroidea	Geometridae	*Scopula ornata*	+	-	-
Drepanoidea	Drepanidae	*Drepana *sp.	+	-	-
Calliduloidea	Callidulidae	*Callidula *sp.	+	-	-
Noctuoidea	Lymantriidae	*Lymantria dispar*	+	-	-
Bombycoidea	Saturniidae	*Aglia tau*	+	-	-
	Bombycidae	*Bombyx mori*	+	-	+
		*Oberthueria caeca*	+	-	+
Lasiocampoidea	Lasiocampidae	*Lasiocampa quercus*	+	-	-
Pyraloidea	Pyralidae	*Pyralis farinalis*	+	-	-

In total, 17 species were used in the further PCR screening in addition to *Maculinea *spp. (Table 2). We also performed PCR with original degenerate CR1 primers (CR1-S and CR1-A) to demonstrate presence of CR1 clade in all studied species. All specimens gave us the positive signals with the original CR1 primers (Figure 5). The products with the expected size, 550 bp in length, were detected by electrophoresis in all reactions. Thus, we can assume that all studied species contain CR1 clade elements in their genomes.

PCR screening with specific primers demonstrated presence of the CR1A family in all Lycaenidae but its lack in all other studied species. PCR products of the expected size were detected in *Scolitantides orion, Shijimaeoides divina*, and *Plebejus argus*. The genera *Scolitantides *and *Shijimaeoides *are the closest relatives of *Maculinea*. It appears that CR1A family is found in Lycaenidae, but is absent from other butterflies even in closely related families Pieridae (*Pieris napi*), Nymphalidae (*Araschnia levana *and *Melitaea phoebe*), and Satyridae (*Erebia theano*). On the other hand, CR1A-like elements could further diverge in other species to the extent that designed primers would be not appropriate for their detection.

The CR1A PCR products were cloned and sequenced for all three species in which CR1A was detected additionally to *Maculinea *spp. Elements of CR1A family from *S. orion *(SoriCR1A), *Sh. divina *(ShdivCR1A), and *P. argus *(PargCR1A) share high similarity with MacCR1A elements. Average similarity of the amino acid sequences was 94.2 % among CR1A-like elements from all investigated Lycaenidae. The newly identified CR1A elements from Lycaenidae butterflies showed 3.5 % (PargCR1A versus MacCR1A) and 2.3 % (SoriCR1A versus MacCR1A) value of divergence (Table 3).

**Table 3:** Amino acid divergences of genes and CR1 non-LTR retrotransposons from *B. mori *and *M. teleius*.

Gene	Length	Amino Acid Divergence (%)
EF-1a	322 aa	
*Bombyx *versus *Maculinea*		2.8
COI	368 aa	
*Bombyx *versus *Maculinea*		14.4
COII	207 aa	
*Bombyx *versus *Maculinea*		15.3
CR1 RT non-LTR retrotransposons		
BmCR1B *Bombyx *versus CR1B *Maculinea*	180 aa	2.7
BmCR1B *Bombyx *versus CR1A *Maculinea*	180 aa	19.9
BmCR1B Bombyx versus OcaCR1B *Oberthueria*	151 aa	4.6
OcaCR1B *Oberthueria *versus CR1B *Maculinea*	151 aa	4.6
OcaCR1B *Oberthueria *versus CR1A *Maculinea*	151 aa	19.6
PargCR1A *Plebejus *versus CR1A *Maculinea*	171 aa	3.5
SoriCR1A *Scolitantides *versus CR1A *Maculinea*	171 aa	2.3
ShdivCR1A *Shijimaeoides *versus CR1A *Maculinea*	171 aa	2.3

Positive results in PCR reaction with CR1B primers were observed for *Maculinea *and representatives of the family Bombycidae: *O. caeca *and *B. mori *(Figure 5). The CR1B products were cloned and sequenced. Surprisingly, the CR1B elements from closely related *O. caeca *(OcaCR1B) and *B. mori *(BmCR1B) showed higher level of amino acid sequence divergence than CR1B retrotransposons from *Maculinea *and *B. mori *(Table 3). Taxa which are most closely related to *Maculinea*, *S. orion *and *Sh. divina*, did not demonstrate a presence of this family in PCR screening (Figure 5). The discontinuous distribution, high sequence similarity between MacCR1B and BmCR1B elements from divergent species and lower similarity between BmCR1B and OcaCR1B elements from closely related species could be the evidences for horizontal transmission for non-LTR retrotransposons from CR1B family.

### Evidence of horizontal transmission

Most examples of horizontal gene transfer in eukaryotes involve transposable elements [35,36]. Such transfers are usually recognized by the presence of very closely related elements in distantly related host taxa. Evolutionary dynamics of transposable elements could sufficiently differ even in closely related taxa. As a result, phylogenetic studies of mobile elements often show incongruence with host species phylogenies. First, transposable elements are multicopy components of genomes. Thus, comparisons of paralogous copies of these elements instead of orthologs along with varying rates of sequence evolution of mobile elements copies are the main sources for phylogenetic incongruence, which could be misidentified as horizontal transmission. Second, random loss of transposable elements from a few taxa, ancestral polymorphism and independent assortment of copies into descendant species, and unequal DNA substitution rates in different species all can lead to incongruence in phylogenetic reconstruction.

Traditionally, horizontal transfer has been implied when highly similar transposable elements have been found in distantly related taxa accompanied by their discontinuous distribution, and such phenomenon could not be explained in terms of vertical inheritance [37-39].

**Figure 6:**
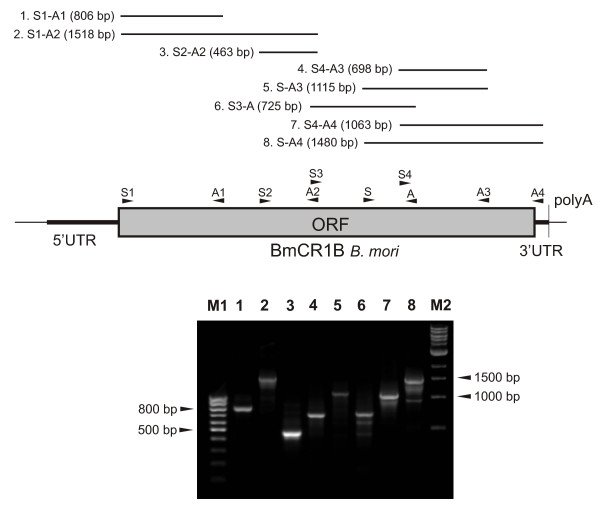
**Scheme of the BmCR1B retroelement and the relative position and size of the fragments amplified by PCR primers in *Maculinea teleius***. Product verified on 1.3% agarose gel and stained with ethidium bromide. Lane M1, 100 bp DNA ladder; lane M2, 1 kbp DNA ladder; number of other lanes correspond to numbers of fragments indicated above.

We found that CR1B family of retrotransposons has discontinuous distribution in butterflies. It was detected in *Maculinea *spp. and two representatives of family Bombycidae: *O caeca *and silkworm *B. mori*. No CR1B-like elements were found in the taxa closely related to *Maculinea*. To extend the analysis, we designed several pairs of primers to cover almost the entire BmCR1B element in PCR amplification. These primer pairs were used to screen *M. teleius *DNA. Products with appropriate size were obtained for all used primer pairs (Figure 6). This is an indirect evidence of high similarity of elements from *B. mori *and *M. teleius *not only in the known sequence of isolated RT fragment but in the entire sequence of BmCR1B and MacCR1B.

By comparing the amino acid sequence divergence for host genes and transposable elements evolving under presumably similar selective pressure the case of horizontal transmission could be detected if divergence among mobile elements is significantly lower than that observed for the proteins encoded by host genes. We compared the partial amino acid and nucleotide sequences of the elongation factor 1 alpha (EF-1α) gene from *Maculinea *spp. and *B. mori *since only this nuclear gene from *Maculinea *was available in databases. It is necessary to note that EF-1α gene is highly conserved in amino acid sequence and is usually used for reconstruction of high-level phylogenies, especially in insects [40-42].

The EF-1α gene comparisons demonstrated almost the same level of amino acid divergence as the mobile elements from CR1B family, 2.8% for EF-1a genes and 2.7% for CR1B elements (Table 3). The nucleotide sequences of EF-1α gene had much lower similarity to each other. Only 83% similarity was detected for EF-1α gene whereas more than 96% similarity showed BmCR1B and MacCR1B non-LTR retrotransposons at DNA level. It seems unlikely that 96% nucleotide and 98.3% amino acid sequence similarity could be selectively or otherwise maintained in retrotransposons that diverged from a common ancestor.

The amino acid sequences of mitochondrial cytochrome oxidase subunit I (COI) and subunit II (COII) genes also were compared among studied species. It seems that RT of CR1B elements is considerably more conserved than compared mitochondrial proteins. We found 14.4% and 15.3% divergence at the amino acid level in COI and COII genes, respectively and only 2.7% average divergence between RT BmCR1B and MacCR1B elements (Table 3).

Since the last common ancestor (LCA) of lepidopteran superfamilies Papilionoidea and Bombycoidea has been estimated to exist 140 million years ago (Mya) [43], it is very difficult to explain the extreme nucleotide and amino acid conservation, together with the discontinuous distribution, without invoking the horizontal transfer of CR1B elements. We also compared the sequences of other insect non-LTR retrotransposons from CR1 and closely related clades for which strong vertical inheritance was suggested (Table 4). Even closely related Jockey elements from *Drosophila melanogaster *and *D. funebris *(LCA estimated at 40 Mya) were more than 24% divergent. The value of amino acid sequence divergence between MacCR1B and BmCR1B was comparable with those among CR1A elements from Lycaenidae butterflies and between CR1B elements from closely related *O. caeca *and *B. mori *(Table 4).

**Table 4:** Amino acid divergences and evolutionary rates in the CR1 and Jockey clades.

Non-LTR retrotransposons	LCA (divergence time in MYA)	Amino Acid Divergence (%)	Evolutionary rate (10^-9^)
CR1 clade			
Q *Anopheles *versus worf *Drosophila*	260	68.2	1.70
T1 *Anopheles *versus worf *Drosophila*	260	66.3	1.52
BmCR1B *Bombyx *versus CR1B *Maculinea*	140	2.7	0.061
SoriCR1A *Scolitantides *versus CR1A *Maculinea*	10	2.3	1.2
BmCR1B *Bombyx *versus OcaCR1B *Oberthueria*	10	4.6	1.7
MteQ *M. teleius *versus Q *Anopheles*	350	60.0	1.29
Jockey clade			
Jockey *D. melanogaster *versus Jockey *D. funebris*	40	24.7	3.55
AMY *B. mori *versus Helena *D. mauritiana*	350	52.4	1.00
JuanA *Aedes *versus Jockey *D. melanogaster*	~250	60.5	1.79
NLR1Cth *Chironomus *versus Jockey *D. melanogaster*	~250	56	1.59
NLR1Cth *Chironomus *versus AMY *B. mori*	350	67.4	1.22

Overall, our data clearly indicate that the high degree of similarity we observed between the *Bombyx mori *BmCR1B and *Maculinea *CR1B is not due to selection. The only reasonable alternative explanation is horizontal transfer.

### Horizontal transmission in non-LTR retrotransposons

Horizontal transfer can be defined as the process by which genes can move between reproductively isolated species. It is not surprising that many examples of horizontal transfer of transposons have been identified in eukaryotes. There are several features of transposon behavior that make them particularly prone to horizontal transfer. Transposable elements have the capacity to insert themselves into the chromosomes of possible vectors and, subsequently, into host chromosomes. Subsequent to transfer, they can spread rapidly throughout a given species, as is evidenced by the rapid spread of P elements in *D. melanogaster *[37]. Most studied cases of horizontal transmission involve DNA transposons [38,39].

Among retrotransposons, several examples of horizontal transfer have been documented between closely related species (mostly among *Drosophila*) for LTR retrotransposons such as *copia *[44,45] and *gypsy *[46]. The possible mechanism of horizontal transfer of LTR retrotransposons could be their ability to ride along on cross-species viral infections. The presence of envelope-like coding sequences makes some of the LTR retrotransposons capable of virus-like particle formation.

In contrast, non-LTR retrotransposons are believed to be inherited exclusively vertically. Extensive phylogenetic and comparative studies dismissed majority of putative horizontal transfer reports for non-LTR retrotransposons [3]. Nevertheless, strong evidence of horizontal transmission that cannot be neglected, were provided by Zupunski et al. (2001) [15] for Bov-B retroelements which have been relatively recently transferred from the ancestral snake lineage (Boidae) to the ancestor of ruminant mammals [15,47]. Thus, one should not exclude a possibility of occasional horizontal transmission events in non-LTR retrotransposons.

The compared Bov-B elements from Squamata and Ruminantia (LCA 310 Mya) showed the same divergence at the amino acid level as that between *Mus *and *Rattus *L1 elements (LCA 15 Mya). The divergence rate in Squamata versus Ruminantia Bov-B was found to be very low in comparison with other elements from the same clade [15].

The slowdown effect on evolutionary rates was also observed in our study. We estimated sequence divergence rates for non-LTR retrotransposons from insects, including those for CR1 and Jockey clades (Table 4). The divergence rates appeared to be almost the same in all comparisons with exception of drosophilid Jockey elements and CR1B elements from butterflies. However, while in the case of Jockey elements from *Drosophila melanogaster *and *D. funebris *the rate was much higher in comparison with other pairs, CR1B retrotransposons from *B. mori *and *Maculinea *spp. showed significantly low rate of divergence. It was much lower than for the elements from closely related species: SoriCR1A from *Scolitantides *and CR1A from *Maculinea*; BmCR1B from *Bombyx *and OcaCR1B from *Oberthueria *(Table 4).

The actual mechanisms of horizontal transfer are still unknown for mobile elements from eukaryotes since it is not possible experimentally to show how the horizontal transmission can occur. Parasites, symbionts, bacteria, or viruses all could be suggested as potential vectors for horizontal transmission. However, only those putative vectors could be under consideration, which have wide host range and access to germ line cells [48]. Since horizontal transfer detected in present study occurred within butterflies and moths, all viruses and parasites common for Lepidoptera could be suspect as possible vectors.

The direction of CR1B retroelements horizontal transfer is also under question. Since it is unlikely that horizontal transfer could occur simultaneously and independently to two distantly related taxa, those elements could hardly be transmitted recently to *B. mori *and genus *Maculinea *from an unknown species. On the other hand CR1B elements were detected not only in *B. mori*, but also in other representative of Bombycidae family – *O. caeca*. It is highly possible that one of the moths from family Bombycidae was a source of the CR1B elements for a common ancestor of *Maculinea *species.

### CR1A and CR1B as potential molecular markers

Insertion events of retrotransposable elements have been recently demonstrated to be powerful tools in phylogenetics and population genetics studies in various organisms [49-51]. Integration of a non-LTR retrotransposon to a new place is an irreversible event. Non-LTR retrotransposons, once inserted in chromosomal DNA, appear to be fixed. Since retrotransposition is thought to be more or less random with respect to the region of insertion, insertions at exactly the same location appear to be unlikely. The most popular transposon-based marker method is the Sequence-Specific Amplification Polymorphism approach (S-SAP), also called "transposon display" [52]. The S-SAP markers were developed for wide range of taxa, in particular in plants [52,53], insects [50], and fungi [54].

CR1A and CR1B non-LTR retrotransposons from *Maculinea *species showed very high homogeneity among studied species, and thus cannot be used for inferring interspecific phylogenies; however, these elements could be used as markers in population genetics studies.

## Conclusion

**Figure 7:**
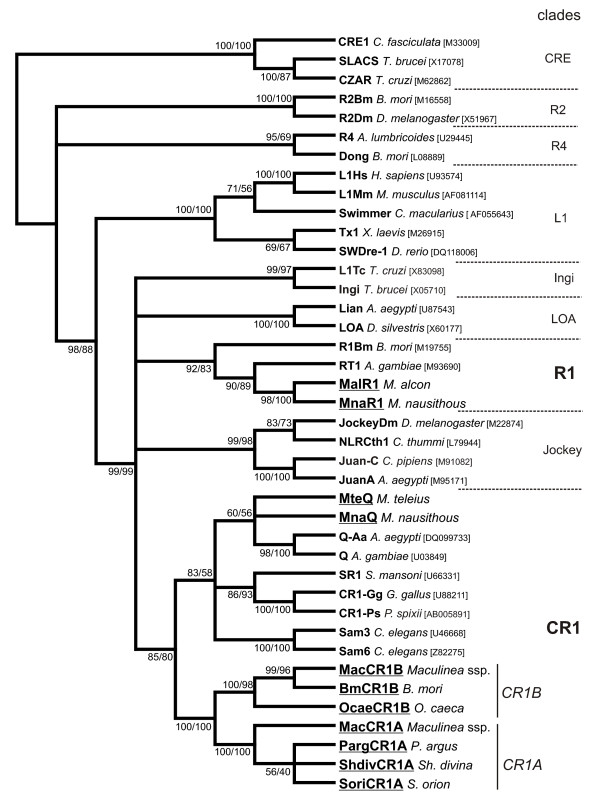
**Neighbour-joining (NJ) phylogeny of RT domains of non-LTR retrotransposons including newly described elements**. The consensus tree is represented. Two bootstrap values correspond to the NJ tree (1000 replications) and maximum likelihood (ML) tree (100 replications). The name of the host species and GenBank accession number is indicated for each non-LTR element.

Our results demonstrated that lycaenid butterflies from the genus *Maculinea*, a model group in the European biodiversity studies, have multiple lineages of elements from CR1 clade. Three families of CR1-like elements coexist in the genomes of *Maculinea *spp. T1Q lineage showed very low similarity to two other lineages, MacCR1A and MacCR1B, whereas MacCR1A and MacCR1B are highly similar to each other and formed a common branch (Figure 7).

Sequences with extremely high similarity to *Maculinea *were identified in genome of silkworm *Bombyx mori*. A non-LTR retrotransposon BmCR1B belonging to CR1B lineage was reconstructed based on short fragments available in databases. The distribution of CR1A and CR1B families was studied among 17 species of Lepidoptera from nine superfamilies. Only representatives of the family Lycaenidae gave positive signal in PCR amplification for the CR1A lineage. None of the studied species showed presence of CR1B family, except *Maculinea *and two moths from family Bombycidae: *Oberthueria caeca *and *Bombyx mori*.

The studied MacCR1B and BmCR1B elements are considerably more conserved than the mitochondrial proteins and have the same level of conservation as EF-1α protein, one of the most conserved proteins in eukaryotes. In summary, the data are consistent with horizontal transfer of CR1B elements between species of Lepidoptera at some point well after the divergence of Bombycoidea and Papilionoidea. The degree of similarity of coding sequences in these elements, their discontinuous distribution, and the results of divergence-versus-age analysis, make it highly unlikely that these sequences diverged at the same time as their host taxa. A presence of the CR1B family in two closely related species from family Bombycidea (*Oberthueria caeca *and *Bombyx mori*) allowed us to suppose that horizontal transmission occurred from Bombycidae to the common ancestor of *Maculinea *species. This study presents a new example of horizontal transmission of non-LTR retrotransposons from CR1 clade.

## Methods

### Species collection and total DNA isolation

Table 1 lists *Maculinea *species used in the present study. *M. teleius, M. nausithous*, and *M. arion *specimens originated from southern Poland. *M. alcon *specimens originated from western Siberia (Novosibirsk, Russia). Taxonomy is given after Als et al. (2004) [16] and Bereczki et al. (2005) [55]. Live *Maculinea *larvae were collected in nature and preserved in 96 % ethanol. Species identification of larval stages was performed using the morphology key by Śliwińska et al. (2006) [56]. Total DNA was isolated from several larvae.

Table 2 lists Lepidoptera species from suborder Ditrysia used in present study and their taxonomy. All material was kindly provided by Dr. Oleg Kosterin (Institute of Cytology and Genetics, Novosibirsk) and Dr. Vladimir Dubatolov (Institute of Animal Systematics and Ecology, Novosibirsk). All listed Lepidoptera, except *Maculinea *spp. and *Bombyx mori*, were collected in nature and stored at -40°C. Detailed label data are available from the authors. Genomic DNA was isolated from the thorax and head of two to four individuals for all species except *Plebejus argus, Calidula *sp. and *Oberthueria caeca*. Total DNA extraction and PCR amplification have been done according to standard techniques [57]. For *B. mori*, the DNA of strain Dazao was used in present study.

### CR1 clade PCR amplification and sequencing

Degenerate PCR primers for CR1 clade of non-LTR retrotransposons were designed by inspection of conserved amino acid sequences in the reverse transcriptase (RT) domains of different published non-LTR retroelements. Primer sequences were: CR1-S= 5'-TATCTTCTTCTCCnggnccngaygg-3' and CR1-A= 5'-CAAAAACACTGCCytgnggnacncc-3', where Y = C + T, and N = A + G + C + T. The designed pair of primers flanked the sequence between conserved regions 0 and 4 of RT domain [3,58]. The length of expected PCR products was about 550 bp.

PCR amplification with degenerate primers was performed using 0.1 μg of genomic DNA in 10-μl volume of 10 mM Tris-HCl (pH 8.9), 1 mM (NH_4_)_2_SO_4_, 4 mM MgCl_2_, 200 μM each of four dNTPs, 0.5 μM primers, and 2.5 units of Taq polymerase. After an initial denaturation step for 3 min at 94°C, the PCR reactions were subjected to 30 cycles of amplification consisting of 30 sec denaturation at 94°C, 42 sec annealing at 52°C, and 1 min extension at 72°C. PCR results were assayed by agarose gel electrophoresis.

The resulting PCR products were directly ligated into a pGEM vector using a pGEM-T-easy cloning kit (Promega) for sequence determination. Clones were amplified by PCR with M13 primers, and 40 ng of the product was used in a 10 μl cycle sequencing reaction with the ABI BigDye Terminator Kit on an ABI 377 DNA sequencer. Sequences were deposited to GenBank under accession numbers [GenBank:DQ822995-DQ823039], [GenBank:DQ836362 - DQ836391] and [GenBank:DQ994657 - DQ994665] (Table 1).

### BmCR1B retrotransposon reconstruction

Nucleotide and protein sequence searches of the GenBank databases with MacCR1A and MacCR1B were performed with BLAST [59] search programs of the NCBI [60]. The following sequence databases were searched at NCBI: nonredundant (NR) and *Bombyx mori *WGS database for Dazao [61] and p50T strains [62]. BmCR1B element sequence was reconstructed by assembling of partial sequences from different contigs (Figure 2). The sequence of the entire BmCR1B element could be found in Supplementary Material (Additional file 2), as well as the list of contigs used for reconstruction (Additional file 3).

Based on the sequence of reconstructed BmCR1B element, we designed PCR primers with the following sequences: BmCR1B-S1 5'-CCCTTTCCTTCCCCACCCC; BmCR1B-S2 5'-TAGCACCTGCGCTGACTCGG; BmCR1B-S3 5'-GGTTCATTGGCATATTCCGC; BmCR1B-S4 5'-GCGGAACGGAGAGTTAAAGTCG; BmCR1B-A1 5'-TCTTCCACATCCGGCACACG; BmCR1B-A2 5'-GTCCAGAGTCGAGTTTCCCGC; BmCR1B-A3 5'-CGCAGTGCCAAAGGATCTAGC; BmCR1B-A4 5'-GAGGCAAACGAGCAAGACGGG.

PCR amplification with designed pairs of primers was performed using 0.1 μg of *M. teleius *genomic DNA in 10-μl volume of 10 mM Tris-HCl (pH 8.9), 1 mM (NH_4_)_2_SO_4_, 2 mM MgCl_2_, 200 μM each of four dNTPs, 0.5 μM primers, and 2.5 units of Taq polymerase. After an initial denaturation step for 3 min at 94°C, the PCR reactions were subjected to 30 cycles of amplification consisting of 30 sec denaturation at 94°C, 42 sec annealing at 54°C, and 1 min extension at 72°C. PCR results were assayed by agarose gel electrophoresis (Figure 6).

### CR1A and CR1B families PCR screening

Based on newly isolated MacCR1A, MacCR1B and BmCR1B elements, specific primers for PCR were designed for each family. The location of primers is shown on Figure 4. Sequences of primers were: for MacCR1A CR1A-S 5'-TCTTTTCGGTTCTCCTACTC and CR1A-A 5'-GCATCAACAGACTGAGTTTCCGAG; for MacCR1B CR1B-S 5'-CTTATTCAGACACTCTTATGTCC and CR1B-A 5'-GAGCATGAGCCGTCTACGA PCR amplification with CR1A and CR1B pairs of primers was performed using 0.1 μg of genomic DNA in 10-μl volume of 10 mM Tris-HCl (pH 8.9), 1 mM (NH_4_)_2_SO_4_, 1.5 mM MgCl_2_, 200 μM each of four dNTPs, 0.5 μM primers, and 2.5 units of Taq polymerase. After an initial denaturation step for 3 min at 94°C, the PCR reactions were subjected to 30 cycles of amplification consisting of 30 sec denaturation at 94°C, 42 sec annealing at 52°C, and 1 min extension at 72°C. PCR results were assayed by agarose gel electrophoresis.

The resulting PCR products were directly ligated into a pGEM vector using a pGEM-T-easy cloning kit (Promega) for sequence determination. Clones were amplified by PCR with M13 primers, and 40 ng of the product was used in a 10 μl cycle sequencing reaction with the ABI BigDye Terminator Kit on an ABI 377 DNA sequencer. Sequences were deposited to GenBank under accession numbers [GenBank:EF591634], [GenBank:EF592105], [GenBank:EF592106], [GenBank:EF592107 ], [GenBank:EF592108].

### Sequence and phylogenetic analysis

Multiple DNA sequence alignment was performed by ClustalW [63] and used for phylogenetic tree construction based on the neighbor-joining (NJ) method, implemented in MEGA 3.0 [65]. Pairwise distances were estimated under Kimura's two parameter model by MEGA 3.0. The amino acid sequences of newly identified RT and RT from GenBank were aligned using ClustalW [63] and edited manually. Phylogenetic analysis was carried out using the NJ method in MEGA 3.0 program [65] and Maximum Likelihood (ML) optimality criteria in PHYML algorithm [66]. For the ML analysis, the appropriate substitution model was calculated using ProTest program, version 1.3 [67]. The best evolutionary models for the analyzed amino-acids data set were rtREV based on the Akaike Information Criterion (AIC) [68] and Blosum62 based on Bayesian Information Criterion (BIC) [69]. Statistical support for the trees was evaluated by bootstrapping: 1000 replications for NJ and 100 replications for the ML [70].

Evolutionary rates were estimated by standard methods [71]. Poisson correction distances (d) were estimated from the equation d = -ln(1 - p), where p represents the proportion of different amino acids. The rate of amino acid substitution (r) was estimated by the standard equation r = d/2T, where T is the divergence time of the last common ancestor (LCA) of the compared species. Amino acid distances used in divergence-versus-age analysis were calculated from sequences of the partial RT domain (~300 aa) using MEGA 3.0 [65].

## Abbreviations

LTR – long terminal repeat

TPRT – target-primed reverse transcription

RT – reverse transcriptase

ORF – open reading frame

RLE – restriction enzyme-like endonuclease

APE – apurinic/apyrimidinic endonuclease

polyA – polyadenine tract

UTR – untranslated terminal region

EF-1α – elongation factor 1 alpha

COI – oxidase subunit I

COII – oxidase subunit II

LCA – last common ancestor

Mya – million years ago

MP – maximum parsimony method

ML – method of maximum likelihood

NJ – neighbor-joining method

S-SAP – Sequence-Specific Amplification Polymorphism

## Authors' contributions

ON participated in the design of the study, carried out the analysis, participated in the sequence analysis and drafted the manuscript. ES participated in insect collecting, and data analysis and interpretation. VF participated in drafting the manuscript. JS contributed to data acquisition. AB participated in the design of the study and coordination. MW participated in the design of the study and has given final approval of the submitted version. All authors read and approved the final manuscript.

## Supplementary Material

Additional file 1 Additional data file 1 is a plain text document entitled ''*Maculinea *TEs alignment used for figure 1'' and contains the nucleotide fragments alignment in MEGA format which was used for phylogenetic tree reconstruction (Fig. 1). Click here for file

Additional file 2 Additional data file 2 is a plain text document entitled "BmCR1B whole sequence with features" and contains the nucleotide sequence of reconstructed BmCR1B element from *B. mori *with annotations. Click here for file

Additional file 3 Additional data file 3 is a word document entitled "contigs used in reconstruction BmCR1B" and contains the table of contigs used in reconstruction of BmCR1B element with GenBank accession numbers and locations of BmCR1B fragments. Click here for file
