# Supplementary material for: CR1 clade of non-LTR retrotransposons from Maculinea butterflies (Lepidoptera: Lycaenidae): evidence for recent horizontal transmission
Source: BMC Evol Biol. 2007 Jun 25;7:93. doi: 10.1186/1471-2148-7-93 (PMC1925062; doi:10.1186/1471-2148-7-93)
Supplement: Additional file 3 — Additional data file 3 is a word document entitled "contigs used in reconstruction BmCR1B" and contains the table of contigs used in reconstruction of BmCR1B element with GenBank accession numbers and locations of BmCR1B fragments. [file 1471-2148-7-93-S3.doc]

| contig | GenBank Acc. No. | fragment of BmCR1B element (bp) | strand | location in contig (bp) |
| --- | --- | --- | --- | --- |
| Ctg011054 | [GenBank:AADK01011054] | 1..3141 | plus | 7676..10812 |
| Ctg006777 | [GenBank:AADK01006777] | 405..3525 | plus | 1..3117 |
| Ctg034636 | [GenBank:AADK01034636] | 1305..3525 | minus | 2956..753 |
| Ctg022133 | [GenBank:AADK01022133] | 1..2381 | plus | 3392..5765 |
| Ctg011753 | [GenBank:AADK01011753] | 1..2261 | minus | 2295..36 |
| Ctg028411 | [GenBank:AADK01028411] | 1507..3524 | plus | 24..2043 |
| Ctg010781 | [GenBank:AADK01010781] | 1561..3525 | minus | 4254..2290 |
| Ctg004626 | [GenBank:AADK01004626] | 1508..3525 | plus | 855..2873 |
| Ctg016714 | [GenBank:AADK01016714] | 1562..3525 | plus | 36..1997 |
| Ctg042860 | [GenBank:AADK01042539] | 1282..3121 | plus | 1..1818 |
| Ctg018950 | [GenBank:AADK01018950] | 5..546 | plus | 6330..6870 |
| Ctg001782 | [GenBank:AADK01001782] | 1..1629 | minus | 1688..62 |
| Ctg017498 | [GenBank:AADK01017498] | 1767..3525 | minus | 7446..5689 |
| Ctg007817 | [GenBank:AADK01007817] | 1553..3525 | minus | 13493..11523 |
| contig517194 | [GenBank:BAAB01124763] | 1..445 | plus | 4301..4743 |
| contig528828 | [GenBank:BAAB01130043] | 5..1193 | plus | 767..431 |

Table 1 (Supplementary material). Reconstruction of BmCR1B element: contigs which were used, their accession numbers, location of BmCR1B fragments.
